# Topoisomerase-I PS506 as a Dual Function Cancer Biomarker

**DOI:** 10.1371/journal.pone.0134929

**Published:** 2015-08-06

**Authors:** Ming Zhao, Ruth A. Gjerset

**Affiliations:** 1 RG Biopharma, 3550 General Atomics Court, San Diego, California, 92121, United States of America; 2 RG Biopharma and Torrey Pines Institute for Molecular Studies, 3550 General Atomics Court, San Diego, California, 92121, United States of America; Peking University Cancer Hospital & Institute, CHINA

## Abstract

Novel biomarkers for cancer diagnosis and therapy selection are urgently needed to facilitate early detection and improve therapy outcomes. We have previously identified a novel phosphorylation site at serine 506 (PS506) on topoisomerase-I (topo-I) and have shown that it is widely expressed in cell lines derived from several cancers, including lung cancer, but is low in cell lines derived from non-cancerous tissues. Here we have investigated how PS506 expression in lung tissue specimens correlates with their malignant status. We find that PS506 expression is significantly elevated in malignant tumors of non-small cell lung cancer (NSCLC) compared to adjacent, non-cancerous lung tissue and benign lung tumors. PS506 expression was up to 6-fold higher in malignant specimens than in paired non-malignant tissue. Using the well-characterized NIH/NCI 60-cell line panel, we correlate the most elevated expression levels of PS506 in lung, ovarian, and colon cancer cells lines with increased sensitivity to camptothecin, a plant alkaloid that targets topo-I. This is consistent with our earlier studies in a smaller sampling of cell lines and with our finding that PS506 increases topo-I DNA binding. Two widely used chemotherapeutic drugs for ovarian and colon cancer, topotecan and irinotecan, respectively, are derived from camptothecin. Irinotecan has also displayed efficacy in clinical trials of NSCLC. Our results suggest that elevated PS506 expression may correlate with clinical chemosensitivity to these agents in ovarian, colon, and NSCLC. PS506 may therefore serve as a biomarker for diagnosis or therapy selection.

## Introduction

New molecular biomarkers for cancer, particularly those relevant to the mechanism of cancer or to cancer therapy, could enhance the power of currently used diagnostic approaches, reduce the risk of over diagnosis, facilitate treatment selection, and greatly improve cancer survival. We have previously identified a novel phosphorylation site on topoisomerase I (topo I) at serine residue 506 (PS506) that is highly expressed in cancer-derived cell lines but is low to undetectable in cell lines derived from normal tissues, making it a possible candidate as a malignancy-associated biomarker for diagnosis [1].

Topo I plays an essential role in DNA metabolism, and is the unique cellular target for irinotecan and topotecan, two widely used chemotherapeutic drugs derived from the plant alkaloid camptothecin (CPT) [2, 3]. By relaxing DNA supercoils that form during DNA replication and transcription, topo I relieves torsional stress on DNA and allows further progression of the replication fork and transcription complex, respectively [4–6]. A basal level of phosphorylation, primarily on serine residues in its N-terminal domain distinct from PS506 is necessary for topo I DNA binding and activity [7]. Increased topo I phosphorylation occurs in many cancer-derived cell lines and correlates with expression of PS506 in the core domain of the protein [8, 9]. We found that PS506 expression also correlates with increased levels of the serine-threonine protein kinase, CK2, and that recombinant topo I can be phosphorylated on serine 506 by purified CK2 [1]. CK2, which is constitutively expressed at low levels in normal cells [10], tends to increase in cancer, and high levels are indicative of poor prognosis [11–15]. In mice, elevated CK2 potentiates pro-oncogenic signaling pathways and collaborates with oncogenes to promote tumors [16–22], suggesting that CK2 may play a fundamental role in cancer by creating an environment conducive to further oncogenic changes. Thus, aberrant expression of PS506 may be symptomatic of the pro-oncogenic environment created by elevated CK2.

The therapeutic relevance of topo I also makes PS506 a potential biomarker for treatment selection. CK2-mediated S506 phosphorylation of basally phosphorylated recombinant topo I generates a topo I with increased DNA binding activity and increased DNA relaxation activity on supercoiled plasmids [1, 8]. We observed that cell lines expressing elevated PS506 levels were often more sensitive to CPT, consistent with the mechanism of action of this drug, which uses the enzymatic activity of topo I to create its lethal effect [1]. Because CPT and related drugs allow for topo I-mediated DNA nicking but inhibit topo I-mediated DNA religation, they cause the DNA strand break to persist and ultimately to form a lethal DNA double-strand break. This mechanism is believed to account for the therapeutic effects of irinotecan and topotecan [2, 23, 24]. The enhanced DNA binding and DNA relaxation activity we observe with the PS506 form of topo I may therefore contribute to clinical responses to irinotecan and topotecan by promoting the formation of DNA double-strand breaks.

In this study we have explored the hypothesis that PS506 expression is a characteristic of malignancy, and that the highest levels of expression correlate with cellular responses to CPT-based drugs. To evaluate the relationship of PS506 to malignancy we have examined tissue specimens of malignant lung tumors, paired non-malignant adjacent lung epithelium, and benign lung tumors. To assess the utility of PS506 as an indicator of responsiveness to CPT-based drugs, we have screened its expression in cell lines from the NCI-60 cell line panel for which CPT sensitivity profiles were available from the National Cancer Institute/Developmental Therapeutics Program (NCI/DTP) data base. The results indicate a highly selective overexpression of PS506 in all malignant lung specimens examined, compared to adjacent non-malignant tissue and benign tumor specimens. In cell lines derived from lung, colon, and ovarian cancers, we find that the highest levels of PS506 expression correlate with increased CPT sensitivity.

## Materials and Methods

### Antibodies

The pAb506P rabbit IgG was generated to a 21-amino acid phosphopeptide (TVGCCS*LRVEHINLHPELKKC, in which phosphoserine 506 is indicated by *), as previously described [1]. Goat anti-topo I IgG, which recognizes the topo I C-terminus, and mouse monoclonal anti-actin were purchased from Santa Cruz Biotechnology (Santa Cruz, CA). Mouse monoclonal anti-tubulin was purchased from Novus Biologicals (Littleton, CO). The secondary antibodies were goat anti-rabbit-horseradish peroxidase (HRP), goat anti-mouse-HRP, and donkey anti-goat-HRP (Santa Cruz Biotechnology). For post-immunoprecipitation Westerns, the primary antibody was detected using Pierce Clean-Blot IP Detection Reagent (Thermo Scientific, Waltham, MA), which recognizes only whole, intact IgG and not the dissociated light or heavy chain subunits.

### Cell line

NCI-H358 human bronchioalveolar non-small cell carcinoma cells (H358), catalogue number CRL-5907, were purchased in May 2011 from the American Type Culture Collection and were mycoplasma-free. Cells were grown at 37°C in 10% CO_2_ in Dulbecco’s Modified Eagles Medium supplemented with 10% newborn calf serum and additives, as previously described [8]. For camptothecin treatment, cells were incubated for 24 hours in the presence of 0.1 or 1 μM camptothecin (Sigma, St. Louis, MO), followed by harvesting and Western analysis as described below.

### Cell pellets

Frozen cell pellets from the NCI 60-cell line panel were obtained from the Division of Cancer Treatment and Diagnosis (DCTD) Tumor repository of the National Cancer Institute (NCI) and were stored at -80°C until use. The cell lines for which cell pellets were received are listed in (S3 Table).

### Tissue specimens

All tissue specimens used in this study were frozen, anonymous, publicly available archival specimens of non-small cell lung cancer with paired non-malignant control tissue, or specimens from benign lung tumors. All specimens were received from the Western Division of the Cooperative Human Tissue Network (CHTN), Vanderbilt University, Nashville, TN. No identifiable private patient or donor information was supplied with the specimens. Since no data was obtained through intervention or interaction with the individual and since no identifiable private information was supplied to us, this research did not constitute human subjects research as defined by CFR 46.102f and the OHRP Guidance on Research Involving Coded Private Information or Biological Specimens, and was granted exemption from review by the Torrey Pines Institute for Molecular Studies IRB.

### Western analyses

Cell pellets (equivalent to ~10^7^ cells/pellet) or two near-confluent 10-cm plates of PBS-washed H358 cells (equivalent to ~2 x 10^7^ cells) were lysed by the addition of 350 μl per pellet or 700 μl per plate of cold RIPA buffer (10 mM TRIS pH 8, 0.15 M NaCl, 0.1% SDS, 1% NP40, 1% deoxycholate) to which complete protease and phosphatase inhibitors (Roche, Nutley, NJ) were added just prior to use, and processed as previously described [8]. Frozen tissue specimens (average weight ~0.3–0.5 g) were homogenized in 3 ml of RIPA buffer on ice using a tissue grinder and then centrifuged to remove debris. The cell and tissue lysates were aliquoted and frozen at -80°C until use. A freshly thawed lysate was used for each gel run.

Lysates (70 μg of protein, except where indicated) were resolved by SDS-PAGE using Criterion 10–20% Tris-HCl precast gels (Bio-Rad Laboratories, Hercules, CA). An equivalent amount of a freshly thawed aliquot of the reference stock of H358 lysate was included in each run as a common control. Following electrophoreses, proteins were transferred to PVDF membranes and immunostained by incubation with primary antibody (1:500 for tubulin; all others at 1:100), followed by appropriate HRP-conjugated secondary antibody (1:1000), and Pierce ECL reagent (Thermo Scientific), followed by exposure to film. Films were scanned using an Alpha Imager and bands were quantified using the accompanying software. PS506 band intensities were normalized to the reference H358 control.

### Immunoprecipitation/Western blotting

Immunoprecipitations were carried out essentially as described [25]. Briefly, cell lysates from exponentially growing H358 cells were prepared in RIPA buffer as described above. Cellular proteins (1–2 mg) were immunoprecipitated by rocking overnight with 50 μl goat anti-topo I (~10 μg). Immunocomplexes were collected by the addition of 20 μl protein AG agarose followed by centrifugation. Immune complexes were dissociated at low pH in the absence of dithiothreitol or β-mercaptoethanol to avoid dissociation of the IgG. Electrophoresis sample buffer was added and samples were subjected to SDS-PAGE as described for Westerns, without prior boiling to further ensure that the IgG remained intact. The Western was processed as described above except that Clean-Blot IP Detection Reagent (Thermo Scientific) was used to detect the primary antibody.

### ELISA analysis

Serine 506-phosphorylated and non-phosphorylated forms of the 21-amino acid topo I peptide described above (see Antibodies section) were synthesized by Biopeptide Co., Inc. (San Diego, CA). Immulon H2B ELISA plates (Thermo Scientific) were coated overnight at 4°C with 100 μl of peptides resuspended at 2 μg/ml in coating buffer (50 mM NaHCO_3_, pH 9). Plates were washed with washing buffer (Tris-buffered saline [TBS] pH 7.5, 0.5% Tween) and blocked with TBS containing 1% bovine serum albumin (BSA). Plates were washed once with washing buffer and then incubated for 1 h with serial dilutions (in duplicate) of pAb506P in TBS/1%BSA, washed again, and incubated for 1 h with goat anti-rabbit-HRP. Following another wash step, plates were incubated with HRP substrate 3,3’,5,5’-tetramethyl benzidine (1-step SLOW TMB ELISA, Thermo Scientific) according to the manufacturer’s instructions. Absorbance at 450 nM was read.

### Alkaline Phosphatase treatment

300 μg of cell lysate prepared in RIPA buffer in the absence of phosphatase inhibitors was treated for 1 h at 37° C with 300 units calf intestinal alkaline phosphatase (Sigma-Aldrich, Inc., St. Louis, MO) in buffer adjusted to 0.1M NaCl, 0.01M MgCl_2_, 0.001M DTT as suggested by the manufacturer. A parallel control lysate prepared in the presence of phosphatase inhibitors was incubated in buffer lacking alkaline phosphatase.

### Statistical analyses

Statistical analyses were carried out using GraphPad® Prism software (GraphPad Software, Inc., La Jolla, CA).

## Results

### Characteristics of pAb506P

**Fig 1:**
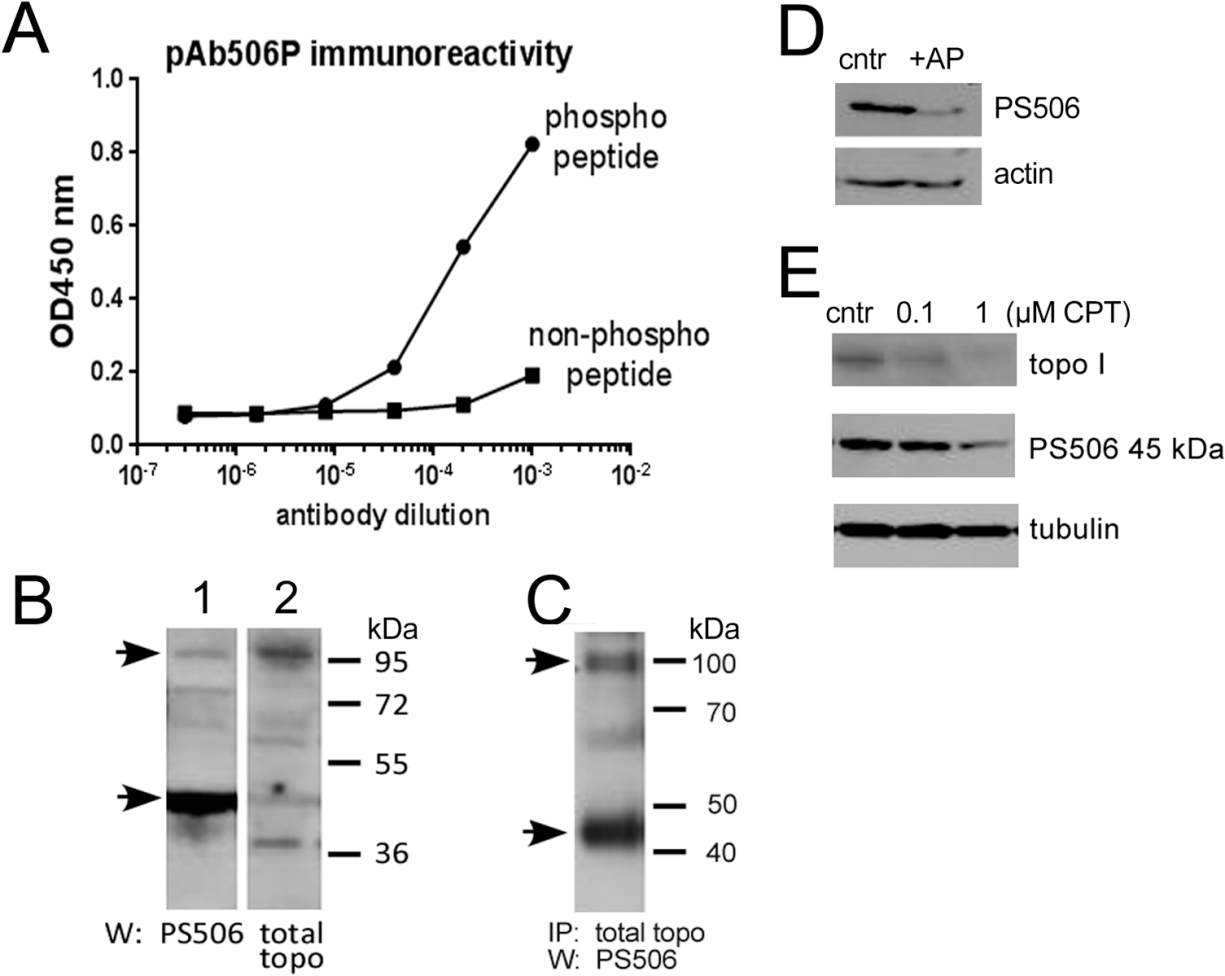
Specificity of pAb506P. **(A)** Comparative titration curves of pAb506P on ELISA plates coated with a topo I peptide surrounding the serine 506 site, either in its phosphorylated or non-phosphorylated form. **(B)** Western analyses of H358 cell lysates (100 μg/lane) probed with pAb506P (lane 1) or with goat anti-topo I (lane 2). Arrows indicate positions of the 45 kDa species and full length topo I. **(C)** Topo I immunoprecipitation (goat anti-topo I C-terminus) followed by pAb506P Western of H358 cell lysates. Lane represents 200 μg starting material. **(D)**Western analysis of PS506 and actin in H358 cell lysates before (cntr) and after treatment with alkaline phosphatase (AP). **(E)** Western analysis of PS506 (using pAb506P), full length topo I (using goat anti-topo I) and tubulin in H358 cells before and after a 24 hr treatment of cells with 0.1 or 1 μM CPT.

The rabbit polyclonal IgG, pAb506P, raised to a PS506-containing peptide unique to topo I, has been previously shown to recognize the 90 KDa cellular topo I in cancer-derived cell lines as well as the PS506-containing form of recombinant topo treated with protein kinase CK2 [1]. The antiserum is highly specific for the phosphorylated form of the immunizing peptide, as shown by the comparative titration curves in peptide ELISA assays (Fig 1A). An additional more prominent pAb506P-reactive species migrating at about 45 kDa on SDS-PAGE/Westerns is also present (Fig 1B, lane 1, arrows indicate positions of the 45 kDa species and full length topo I in H358 NSCLC cell lysates). A topo I species of similar size has been reported in Jurkat leukemic cells [26]. The 45 kDa species appears as a minor band on blots probed with a goat anti-topo I (Fig 1B, lane 2), and can be immunoprecipitated from H358 cell lysates with goat anti-topo I (Fig 1C), indicating that it shares immunoreactivity with full-length topo I but is likely to be a minor species. The weaker reactivity of full length topo I with pAb506P may indicate that it is not fully phosphorylated at this site. pAb506P reactivity is reduced following treatment of H358 cell lysates with alkaline phosphatase, confirming that it represents a phosphorylated species (Fig 1D). Both full-length topo I and the 45 kDa PS506-positive species are selectively down regulated following treatment with camptothecin (CPT), under conditions where the tubulin control remains unchanged, indicating that full length topo I and the 45 kDa species are regulated similarly in response to CPT (Fig 1E). Topo I is the unique target of CPT, and has previously been shown to be specifically down-regulated in CPT-treated cells [27]. Taken together, the results suggest that the 45 kDa species is likely to be a phosphorylated degradation product of topo I. Because it is the primary species detected in cell lysates, we evaluated its expression in tissue specimens.

### PS506 expression in malignant, benign, and normal lung tissue

pAb506P was used to screen PS506 expression in anonymous specimens of non-small cell lung tumors (carcinomas, adenomas) and non-malignant lung tissue (as paired specimens from the same patient), as well as benign lung tumors. Specimens were obtained from the Western Division of the Cooperative Human Tissue Network (CHTN) and are listed with their descriptions in (S1 Table. Characteristics of tumor/non-tumor pairs (provided by CHTN); S2 Table. Characteristics of benign tumors (provided by CHTN)). Tissue homogenates prepared from the paired non-small cell lung tumor and non-malignant lung tissue, and benign lung tumor specimens were analyzed by SDS-Page/Western for the presence of PS506, and levels were quantified by digital analysis of band intensities. To ensure standardization across different gels, we prepared a single lysate of H358 cells, which was frozen in aliquots. A freshly thawed aliquot of this lysate was included in each gel run as a reference standard.

**Fig 2:**
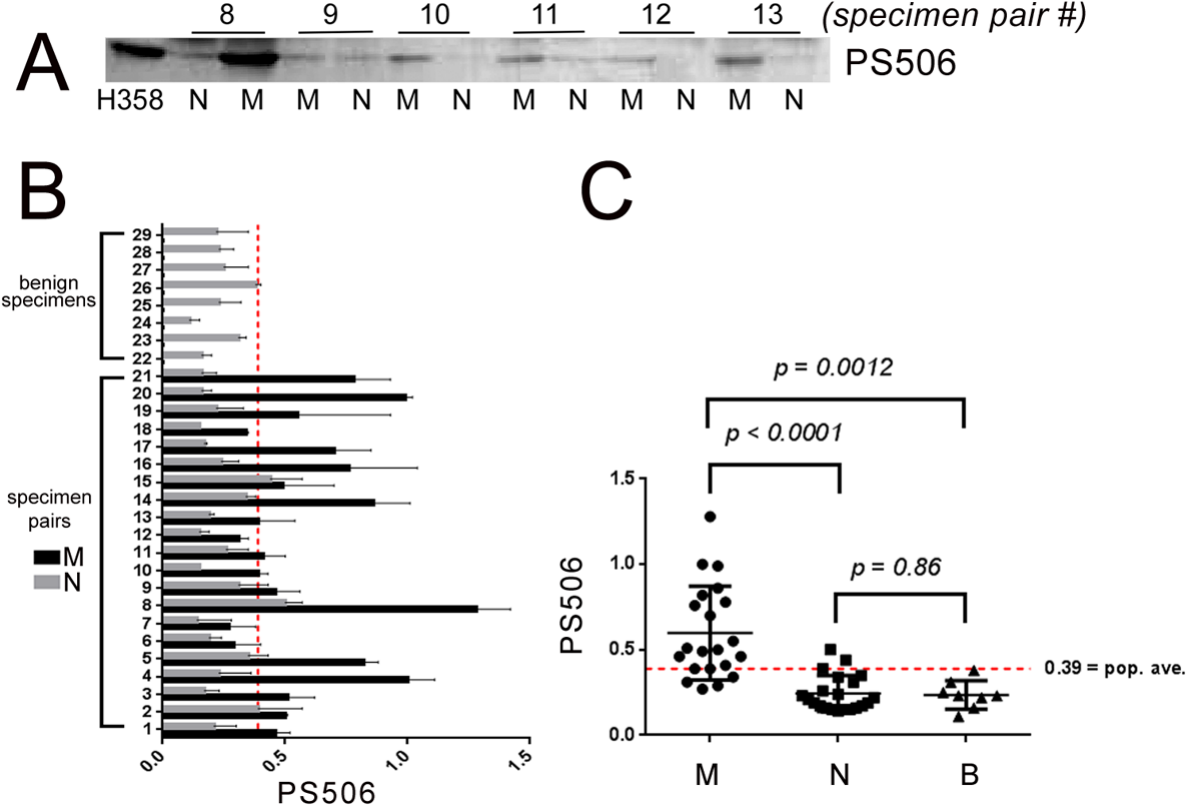
Expression of PS506 in malignant, non-malignant, and benign tumor tissues. **(A)** Representative PS506 Western blot of specimens of NSCLC and their paired non-malignant specimens (specimen pairs 8–13). H358 is the reference control for quantification of all PS506 blots. Tables 1 and 2 list the specimens analyzed and quantification of PS506 levels relative to H358. **(B)** Bar graph of the PS506 levels shown in Tables 1 and 2, grouped as paired malignant/non-malignant specimens and benign tumors. **(C)** Scatter plot of PS506 levels from Tables 1 and 2, grouped as malignant (M), non-malignant (N), and benign (B) tumors. The p values were calculated by an unpaired t-test.

Fig 2A shows a representative Western blot for PS506 in matched malignant/non-malignant specimen pairs 8–13, together with the H358 reference standard. PS506 levels relative to H358 were similarly assessed for all 21 matched pairs plus 8 benign tumors and the results were verified in a second independent experiment. The averages and standard deviations for the two experiments are listed in Table 1 (matched pairs) and Table 2 (benign tumors) and plotted in Fig 2B. In all 21 malignant/non-malignant specimen pairs, PS506 expression was elevated in the malignant specimen. Sixteen of the 21 (76%) malignant specimens expressed about 2-6-fold more PS506 than did the non-malignant paired specimen, with the average being ~3-fold higher. Fifteen of the 21 (71%) malignant specimens expressed PS506 at levels above the overall average of 0.39 for all specimens (red dotted line in Fig 2B). Importantly, none of the 8 benign specimens and only 2 of the 21 paired non-malignant specimens (for a total of 2/29 [7%]) expressed PS506 above this average level. Thus, a malignant specimen was >10 times more likely than a non-malignant specimen to express PS506 at greater than the average level for all specimens.

**Table 1:** PS506 levels relative to H358 control (specimen pairs).

specimen pair #	malignant		Paired non-malignant	
	ave	stdev	ave	stdev
1	0.46	0.06	0.21	0.09
2	0.50	0.01	0.39	0.18
3	0.51	0.11	0.17	0.06
4	1.00	0.11	0.23	0.13
5	0.82	0.06	0.35	0.08
6	0.29	0.11	0.19	0.05
7	0.27	0.11	0.14	0.14
8	1.28	0.14	0.50	0.07
9	0.46	0.10	0.31	0.12
10	0.39	0.04	0.15	0.00
11	0.41	0.09	0.26	0.09
12	0.31	0.04	0.15	0.04
13	0.39	0.15	0.19	0.02
14	0.86	0.15	0.34	0.04
15	0.49	0.21	0.44	0.13
16	0.76	0.28	0.24	0.07
17	0.70	0.15	0.17	0.01
18	0.34	0.01	0.15	0.00
19	0.55	0.38	0.22	0.11
20	0.99	0.03	0.16	0.04
21	0.78	0.15	0.16	0.06

**Table 2:** PS506 levels relative to H358 control (benign specimens).

specimen #	benign	
	ave	stdev
22	0.16	0.04
23	0.31	0.03
24	0.11	0.04
25	0.23	0.09
26	0.38	0.02
27	0.25	0.10
28	0.23	0.06
29	0.22	0.13

The scatter plot in Fig 2C shows PS506 levels in the three sets of specimens: the 21 paired malignant (M) and non-malignant (N) tissues, and the 8 benign tumor tissues (B). The average PS506 levels (indicated by the black lines in each set) are 0.60 (malignant), 0.24 (non-malignant paired), and 0.24 (benign). Differences in mean PS506 levels between sample sets were evaluated by an unpaired, two-tailed t-test. As indicated in Fig 2C, the difference between malignant tissue and non-malignant paired tissue was highly significant (*p*<0.0001), and the difference between malignant tissue and benign tumor tissue was significant (*p* = 0.0115). No significant difference was observed between PS506 levels in benign and non-malignant tissues (*p* = 0.86). These results indicate that PS506 expression is strongly associated with malignancy.

### PS506 expression and CPT sensitivity

In an earlier study examining PS506 levels in a variety of cancer cell lines, we found that the highest PS506 levels were present in cell lines with the greatest sensitivity to the topo I-targeted plant alkaloid, CPT, with differences of about 2–3-fold between CPT-sensitive and-resistant cell lines [1]. To extend these observations, we evaluated relative PS506 levels among the NCI 60-cell line panel, available as frozen cell pellets from the Division of Cancer Treatment and Diagnosis Tumor repository of the NCI. The panel includes cell lines derived from a variety of tumor types, including leukemia, non-small cell lung cancer, colon cancer, melanoma, ovarian cancer, renal carcinoma, prostate cancer, breast cancer, and central nervous system cancers. Because the cell lines have been extensively characterized by the NCI for sensitivity to CPT and other experimental and established chemotherapeutic drugs, they provide a highly standardized resource with which to correlate a potential biomarker with chemosensitivity [28].

Cell lysates from the frozen cell pellets were evaluated for PS506 expression by SDS-PAGE/Western in the same manner as the tissue samples. Bands were quantified digitally and PS506 levels relative to the H358 control value were verified in a second independent experiment and averaged. For the 42 cell lines for which CPT sensitivity data were available, we found that PS506 levels were distributed over a broad range, as was observed for the tumor tissue specimens. The average level of PS506 in all 42 cell lines was 0.37 relative to the H358 common control; a value considerably higher than the average value of 0.24 observed for non-malignant paired tissue or benign tumors (Fig 2). We next compared the relative PS506 values of the 42 cell lines with their sensitivity to CPT using the NCI/Developmental Therapeutics Program (DTP) % growth values established with a single high dose (10 μM) CPT assay [29]. In this assay, a negative percentage growth value indicates loss of starting cell mass due to cell death, whereas a positive value indicates the percentage of untreated cell growth, as described on the DTP website [28].

**Fig 3:**
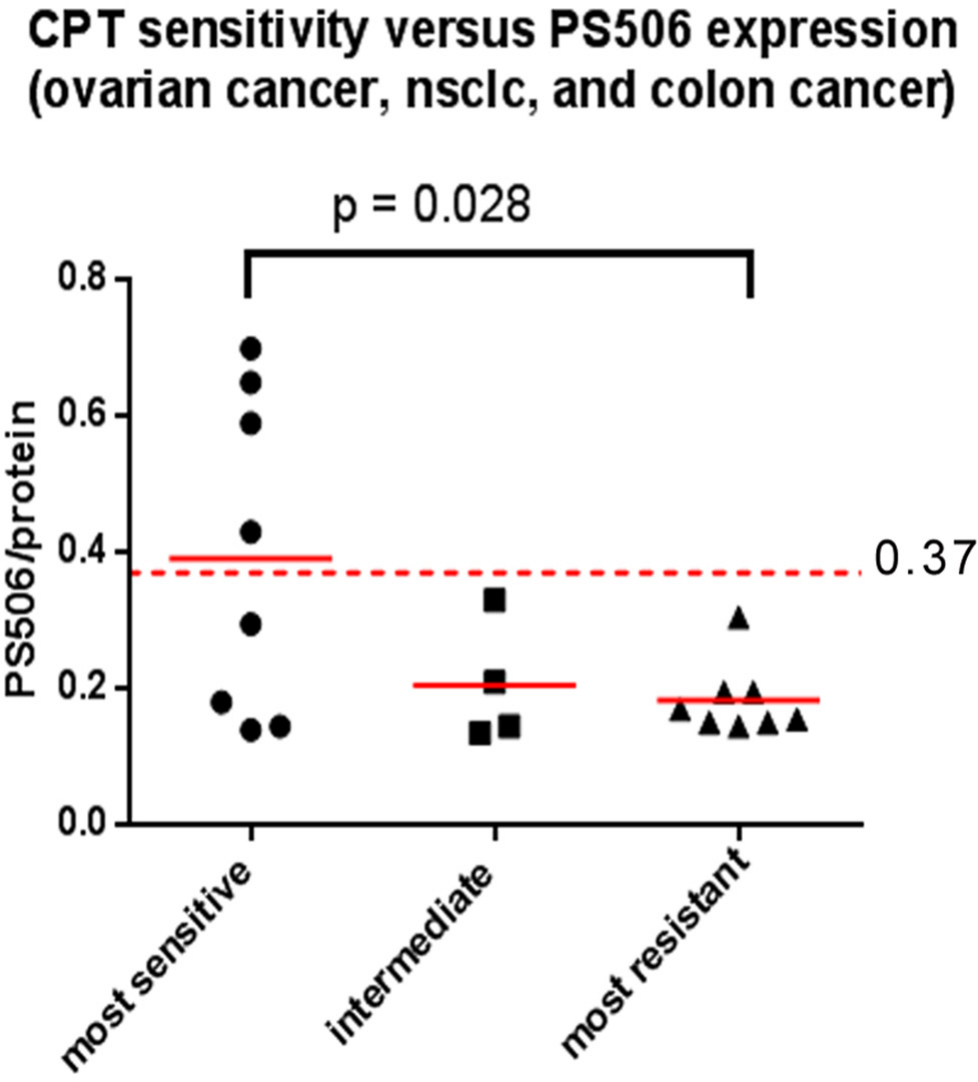
Correlation of CPT sensitivity and PS506 expression in ovarian cancer, NSCLC, and colon cancer. Scatter plot of relative PS506 levels in cell lines representing the 40% most CPT sensitive, the 40% most CPT resistant, and the 20% with intermediate sensitivity among the set of ovarian cancer, colon cancer, and NSCLC cell lines. The red dotted line at 0.37 shows the average PS506 level in the cell lines for which CPT sensitivity data were available. The p value was calculated by an unpaired t-test.

We observed a correlation between PS506 expression and CPT sensitivity in a grouping of 20 non-small cell lung, colon, and ovarian cancer cell lines, representing three cancers for which CPT-based therapies are either FDA-approved (colon, ovarian) or are being evaluated in ongoing clinical trials (non-small cell lung cancer) [30, 31]. Table 3 lists these cell lines, the average PS506 level from the two experiments (relative to H358 cells), and the CPT sensitivity data available on the NCI/DTP web site [28]. Fig 3 shows a scatter plot of the relative PS506 values for the three subgroups defined by their CPT sensitivity: the 8 most sensitive (40%), the 8 most resistant (40%), and the remaining 4 with intermediate sensitivity (20%). The relative PS506 level decreased according to CPT sensitivity from 0.39 (most sensitive) to 0.21 (intermediate sensitivity) to 0.18 (resistant). An unpaired, two-tailed t-test revealed a significant difference (*p =* 0.028) in the average PS506 level between the most sensitive and the most resistant cell lines, consistent with our earlier studies with a smaller sampling of cell lines [1]. These data suggest that for lung, colon, and ovarian cancers, expression of PS506 is one determinant of sensitivity to CPT-based drugs. Importantly, half of the cell lines designated as CPT sensitive–but none of those with intermediate CPT sensitivity or CPT resistance–expressed PS506 at a level exceeding the average of 0.37 for all 42 cell lines.

**Table 3:** CPT sensitivity and PS506 levels in cells lines.

CPT sensitivity	% growth	cell line	relative PS506*	stdev	Cancer type
most sensitive (ave PS506 = 0.39)	-62	IGR-OV1	0.30	0.18	ovarian
	-54	NCI-H226	0.18	0.04	nsclc
	-49	NCI-H522	0.14	0.09	nsclc
	-42	KM12	0.70	0.38	colon
	-38	COLO-205	0.59	0.07	colon
	-36	OVCAR-4	0.15	0.05	ovarian
	-34	OVCAR-3	0.65	0.18	ovarian
	-32	HT29	0.43	0.24	colon
intermediate sensitivity (ave PS506 = 0.21)	-27	NCI-H23	0.14	0.02	nsclc
	-26	NCI-H460	0.15	0.04	nsclc
	-21	A549	0.24	0.14	nsclc
	-19	HCT-15	0.33	0.07	colon
most resistant (ave PS506 = 0.18)	-19	NCI-H322M	0.17	0.03	nsclc
	-13	HCT-116	0.20	0.01	colon
	-10	HOP-62	0.31	0.11	nsclc
	-2	SW-620	0.15	0.01	colon
	-2	OVCAR-5	0.15	0.07	ovarian
	7	SK-OV-3	0.20	0.05	ovarian
	13	EKVX	0.16	0.04	nsclc
	13	NCI/ADR-RES	0.15	0.01	ovarian

* mean of 2 independent analyses, run with same internal standard

We also compared PS506 levels with CPT sensitivity for the larger group of 42 cell lines representing a broader range of cancer types (see: S3 Table. CPT sensitivity and PS506 levels in cell lines from NCI 60 cell line panel). Here, too, we observed a positive association between PS506 levels and CPT sensitivity, with the most resistant cell lines expressing low PS506 levels (40% of lines, mean PS506 level = 0.34) and the most sensitive cell lines expressing higher PS506 levels (40% of lines, mean PS506 level = 0.41), consistent with a model in which PS506 contributes to CPT sensitivity. In the analysis of this larger group, the differences in mean values did not reach statistical significance, however (*p* = 0.43), possibly due to the contribution of cellular factors independent of topo I, such as CPT uptake, intracellular CPT metabolism and distribution, and the cellular response to DNA damage, all of which can affect the outcome of CPT treatments [32]. Nevertheless, the results of this screening support the notion that PS506 levels may be used for therapy selection for certain cancers.

## Discussion

This study demonstrates that expression of PS506 is malignancy-specific, and that the highest levels of PS506 expression in cancer cell lines of lung, colon, and ovarian cancer origin correlate with increased sensitivity to CPT. PS506 levels were elevated in all 21 malignant specimens of non-small cell lung cancer compared to paired non-malignant tissue and to 8 benign lung tumor specimens. In the majority of specimen pairs, the increase in PS506 expression in malignant versus non-malignant tissue ranged from 2-fold to >6-fold. The two non-malignant groups of specimens (21 non-malignant tissues and 8 benign tumor tissues), were not significantly different in PS506 expression. While the absolute levels of PS506 expression in malignant tumor specimens was variable, 71% of malignant tumors displayed PS506 levels above the population average (all specimens combined, i.e., malignant + non-malignant), while only 7% of non-malignant specimens expressed PS506 above the population average.

When we evaluated the NCI-60 cell line panel, we also found that the average PS506 expression level across the cell lines was higher than that of the non-malignant tissue specimens (0.37 in cell lines versus 0.24 in non-malignant specimens). We analyzed the correlation between PS506 expression with cellular sensitivity to the topo I-targeted drug CPT, using CPT sensitivity data available on the NCI/DTP website. In lung, colon, and ovarian cancer cell lines, representing three cancers for which CPT-derived chemotherapeutic drugs are approved or are under clinical testing, we found that the 40% most CPT-sensitive cell lines expressed significantly higher levels of PS506 than did the 40% most resistant cell lines, consistent with our earlier observations in a smaller sampling of available cell lines [1]. Intermediate levels of PS506 expression were observed in the 20% of cell lines with intermediate CPT sensitivity. These results suggest that PS506 may serve, in combination with other clinical factors, to facilitate decision-making for the treatment of patients with the CPT-derived drugs, irinotecan and topotecan. The correct choice of drug is crucial to the success of therapy. This is particularly true of second line therapy, as a failed treatment can result in a declining physical condition of the patient and increases the likelihood that further treatments will be unsuccessful. There are presently no reliable predictive tests for sensitivity to CPT-derived drugs. The application of PS506 as an aid for therapy selection would constitute a critical step toward achieving more individualized treatment regimens.

The development of blood-based assays for PS506 could potentially provide a diagnostic assay for early detection or monitoring of lung cancer or other cancers. Several circulating protein markers are currently in clinical use for certain cancers, primarily for follow-up. These include CA125 for ovarian cancer, CA19-9 for pancreatic cancer, CEA for colon cancer, and PSA for prostate cancer (reviewed in [33]). CYFRA21-1, a cytokeratin marker for epithelial cells in plasma or blood, has been associated with non-small cell lung cancer and other epithelial cancers, but has highly variable sensitivity or specificity for malignancy. A recent report describes an ELISA assay for thymidine kinase in blood specimens for lung cancer detection, [34]. Given that topo I is one of the 10–25% most abundant of cellular proteins in cells and in plasma [35], a similar test for PS506 is likely to be feasible and sensitive. In the case of lung cancer, a sputum-based assay may also be possible, as cancer cells are present in sputum from lung cancer patients. Several other candidate markers have been identified [36, 37]. However, no biomarker has yet been shown to have the necessary sensitivity, specificity, and reproducibility to be validated for routine clinical use, and additional biomarkers are needed.

The possibility that PS506 may be causally linked to cancer greatly increases its interest as a biomarker. Because topo I possesses DNA nicking/religating activity, its tight regulation is critical to avoid aberrant DNA nicking that could destabilize the genome and promote malignant progression [38]. Ectopically overexpressed topo I is recombinogenic in yeast and bacteria and may therefore promote DNA rearrangements and other forms of genome instability in mammalian cells as well [39, 40]. This possibility is supported by evidence that topo I is essential for chromosome breakage at common fragile sites (CFSs), where DNA rearrangements commonly occur in cancer [41, 42]. One well-characterized CFS, FRA3B, is frequently rearranged in lung cancer [43]. Because the PS506 form of topo I has increased DNA association and activity, this raises the possibility that the overexpression of the PS506 epitope in cancer cells predisposes to genome instability, a hallmark of cancer.

A 45 kDa topo I species associated with chemical- or heat-induced necrosis has also been reported in Jurkat leukemia cells [26]. Detection of the 45 kDa species does not appear to be dependent on necrosis in our study, as it was broadly detected in all of the NCI-60 cell lines examined as well as in a culture of H358 cells in which no necrosis was evident. The presence of this species may therefore be a manifestation of the malignant process, possibly an aberrant phosphorylation resulting from overexpression of CK2, a housekeeping kinase that can target serine 506 and is often expressed at higher levels in malignancy.

Furthermore, this study raises a number of interesting questions that warrant further investigation, including the biological relevance of PS506, its role in malignancy, and the basis for its differential appearance in malignant versus normal tissue.

## Supporting Information

S1 Table Characteristics of tumor/non-tumor pairs (provided by CHTN). (DOCX)

S2 Table Characteristics of benign tumors (provided by CHTN). (DOCX)

S3 Table CPT sensitivity and PS506 levels in cell lines from NCI 60 cell line panel. (DOCX)

S1 Fig Original Western blots in Fig 2. (TIF)

S2 Fig Original Western blots in Fig 3. (TIF)

S3 Fig Original Western blots in Fig 1. (TIF)
