# Supplementary material for: Topoisomerase-I PS506 as a Dual Function Cancer Biomarker
Source: PLoS One. 2015 Aug 6;10(8):e0134929. doi: 10.1371/journal.pone.0134929 (PMC4527781; doi:10.1371/journal.pone.0134929)
Supplement: S1 Table — (DOCX) [file pone.0134929.s001.docx]

| **S1 Table. Characteristics of tumor/non-tumor pairs**  **(provided by CHTN)** | | |
| --- | --- | --- |
| **Specimen pair #** | **Specimen type** | **Age/sex/race** |
| 1 | 1) malignant lung carcinoma/adenocarcinoma  2) normal lung | 63/F/W |
| 2 | 1) malignant lung carcinoma/squamous cell  2) normal lung | 59/F/W |
| 3 | 1) malignant lung carcinoma/squamous cell  2) normal lung | 55/F/W |
| 4 | 1) malignant lung carcinoma/adenocarcinoma  2) normal lung | 71/F/W |
| 5 | 1) malignant lung carcinoma/squamous cell  2) normal lung | 71//M/W |
| 6 | 1)malignant cell carcinoma/mucinous adenocarcinoma  2) normal lung | 46/F/W |
| 7 | 1) malignant lung carcinoma/adenocarcinoma  2) normal lung | 67/M/W |
| 8 | 1) malignant lung carcinoma/squamous cell  2) benign lung normal | 71/M/W |
| 9 | 1) malignant lung carcinoma/squamous cell  2) normal lung | 76/M/W |
| 10 | 1) malignant lung carcinoma/adenocarcinoma  2) normal lung | 71/F/W |
| 11 | 1) malignant lung carcinoma/squamous cell  2) normal lung | 61/F/B |
| 12 | 1) malignant lung carcinoma/adenocarcinoma  2) normal lung | 76/F/W |
| 13 | 1) malignant lung carcinoma/squamous cell  2) normal lung | 73/F/W |
| 14 | 1) malignant lung carcinoma/squamous cell  2) normal lung | 75/F/W |
| 15 | 1) malignant lung carcinoma/squamous cell  2) normal lung | 68/F/W |
| 16 | 1) malignant lung carcinoma/squamous cell  2) normal lung | 64/M/W |
| 17 | 1) malignant lung carcinoma/adenocarcinoma  2) normal lung | 72/F/W |
| 18 | 1) malignant lung carcinoma/adenocarcinoma  2) diseased lung (emphysema) | 57/F/W |
| 19 | 1) malignant lung carcinoma/adenocarcinoma  2) diseased lung (emphysema) | 70/F/W |
| 20 | 1) malignant lung  2) normal lung | 82/F/W |
| 21 | 1) malignant lung carcinoma  2) normal lung | 69/F/W |
