# Supplementary material for: Topoisomerase-I PS506 as a Dual Function Cancer Biomarker
Source: PLoS One. 2015 Aug 6;10(8):e0134929. doi: 10.1371/journal.pone.0134929 (PMC4527781; doi:10.1371/journal.pone.0134929)
Supplement: S2 Table — (DOCX) [file pone.0134929.s002.docx]

| **S2 Table. Characteristics of benign tumors**  **(provided by CHTN)** | | |
| --- | --- | --- |
| **Sample #**  **(see Fig.2)** | **Specimen type** | **Age/sex/race** |
| 22 | Benign lung solitary fibrous tumor | 55/F/W |
| 23 | Benign lung hamartoma | Not known |
| 24 | Benign lung | 64M/W |
| 25 | Benign lung solitary fibrous tumor | 65/M/W |
| 26 | Benign lung solitary fibrous tumor | 65/M/W |
| 27 | Benign lung solitary fibrous tumor | 72/F/W |
| 28 | Benign lung solitary fibrous tumor | 65/M/W |
| 29 | Benign lung solitary fibrous tumor | 68/M/W |
