# Supplementary material for: Topoisomerase-I PS506 as a Dual Function Cancer Biomarker
Source: PLoS One. 2015 Aug 6;10(8):e0134929. doi: 10.1371/journal.pone.0134929 (PMC4527781; doi:10.1371/journal.pone.0134929)
Supplement: S3 Table — (DOCX) [file pone.0134929.s003.docx]

**S3 Table**

**CPT sensitivity and PS506 levels in cell lines from NCI 60 cell line panel**

* CPT sensitivities are percent growth in a single CPT dose assay and are taken from the NCI/DTP web site.
