# Supplementary figures and images for: Topoisomerase-I PS506 as a Dual Function Cancer Biomarker
Source: PLoS One. 2015 Aug 6;10(8):e0134929. doi: 10.1371/journal.pone.0134929 (PMC4527781; doi:10.1371/journal.pone.0134929)

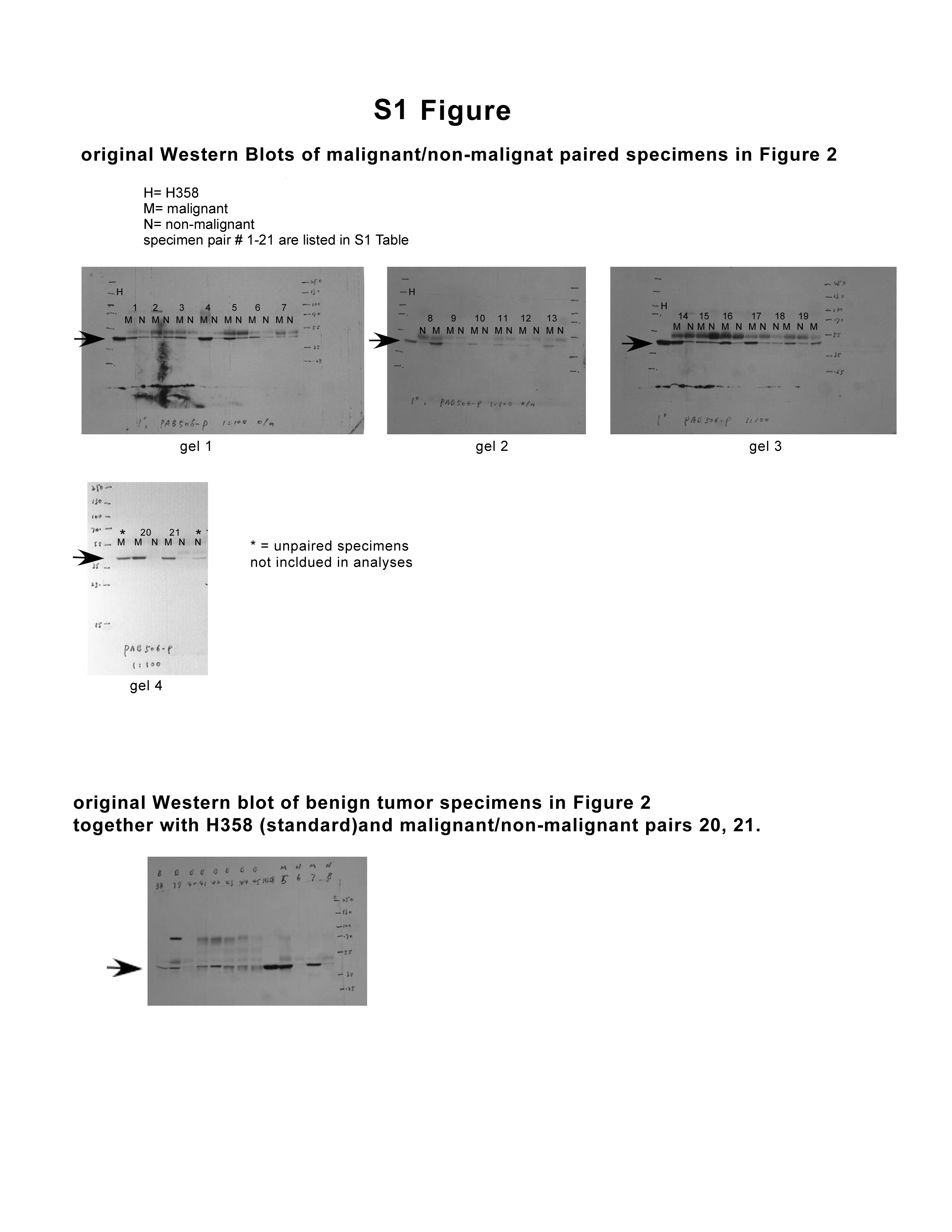

Supplement: S1 Fig — (TIF) [file pone.0134929.s004.tif]

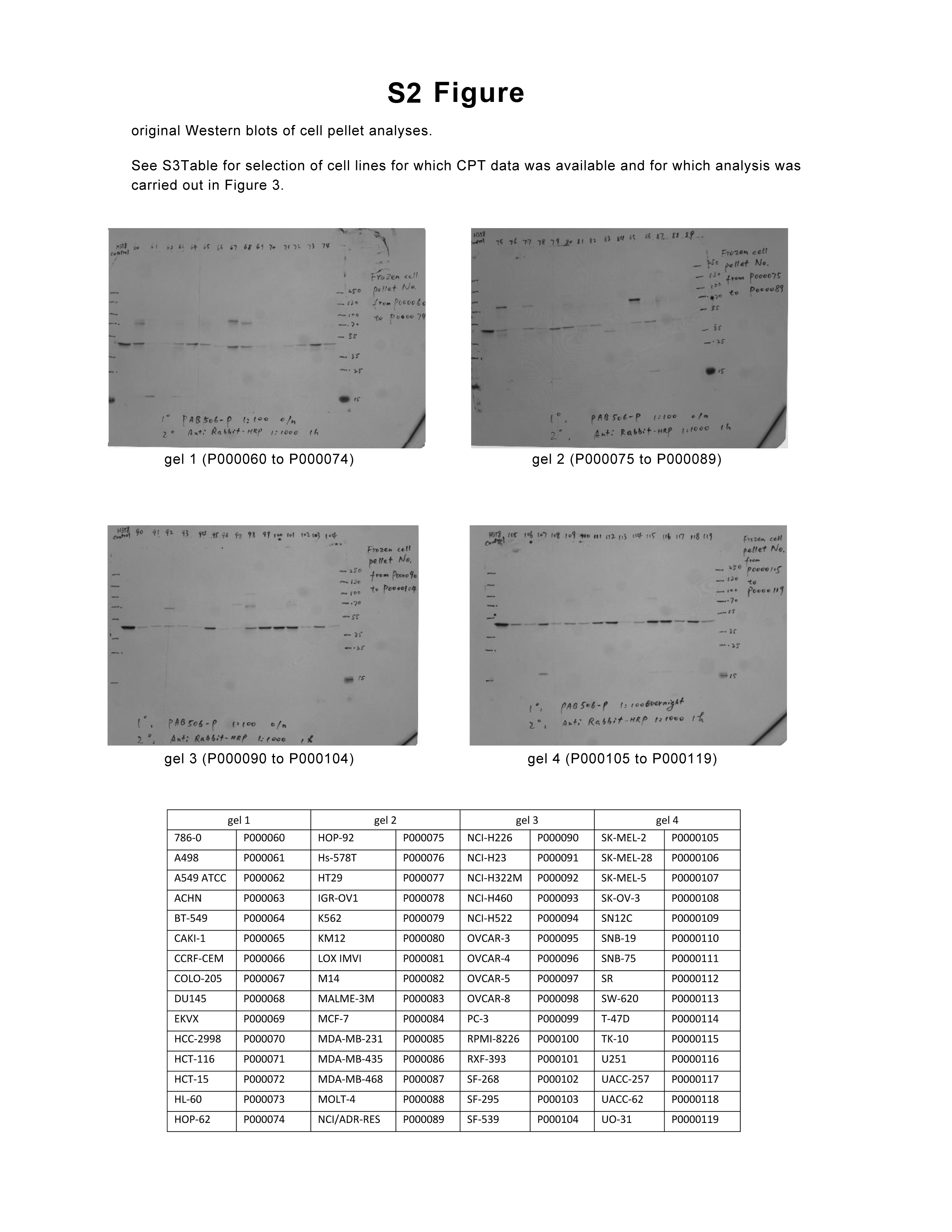

Supplement: S2 Fig — (TIF) [file pone.0134929.s005.tif]

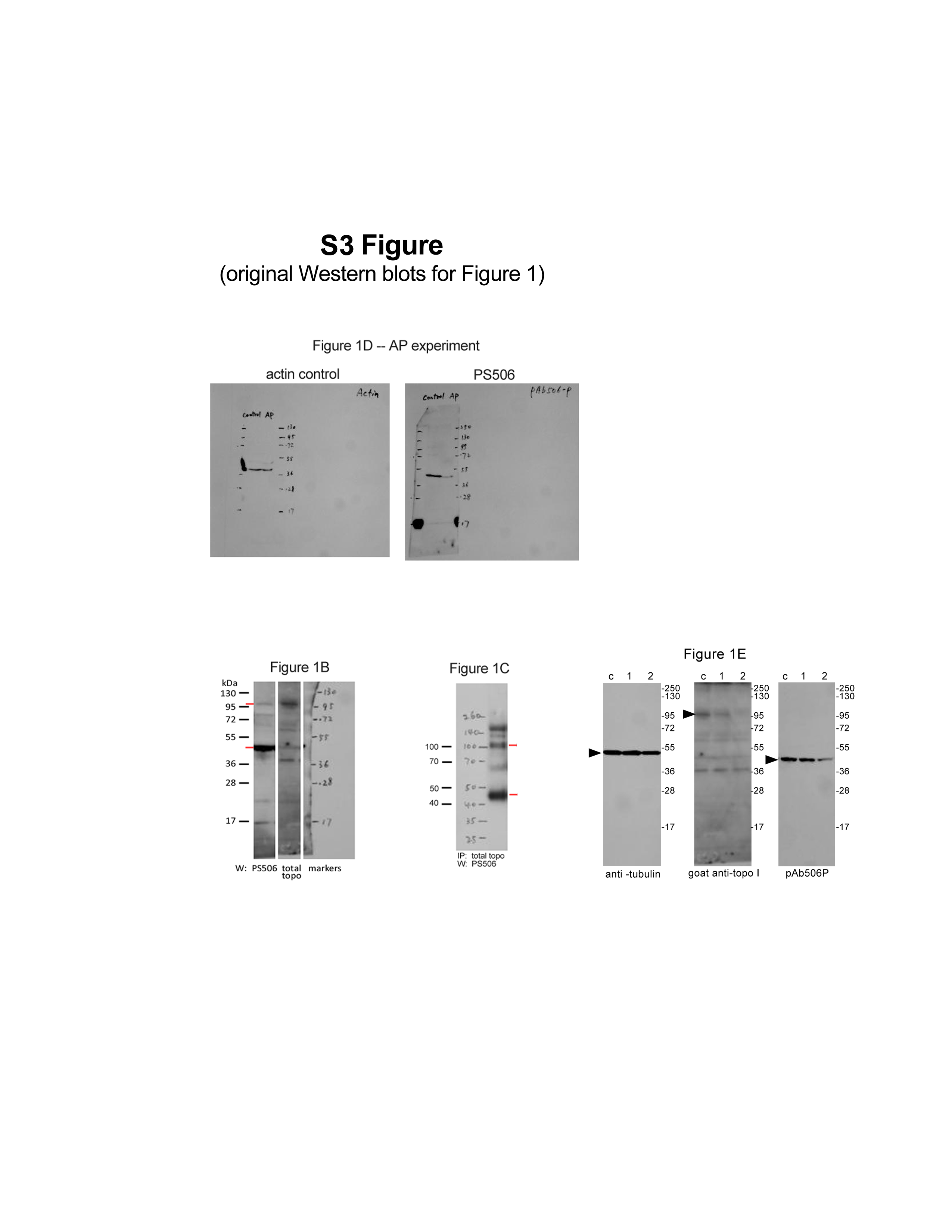

Supplement: S3 Fig — (TIF) [file pone.0134929.s006.tif]
